# Bandwidth-enhanced and Wide-angle-of-incidence Metamaterial Absorber using a Hybrid Unit Cell

**DOI:** 10.1038/s41598-017-14792-0

**Published:** 2017-11-01

**Authors:** Toan Trung Nguyen, Sungjoon Lim

**Affiliations:** School of Electrical and Electronics Engineering, Chung-Ang University, Heukseok-Dong, Dongjak-Gu, 156-756 Republic of Korea

## Abstract

In this paper, a bandwidth-enhanced and wide-angle-of-incidence metamaterial absorber is proposed using a hybrid unit cell. Owing to symmetric unit cells, high absorptivity is maintained for all polarization angles. A circular-sector unit cell enables high absorptivity under the oblique incidence of both transverse electric (TE) and transverse magnetic (TM) modes. To enhance the bandwidth, we introduced a hybrid unit cell comprising four circular sectors. Two sectors resonate at 10.38 GHz, and two resonate at 10.55 GHz. Since the two absorption frequencies are near each other, the bandwidth increases. The proposed idea is demonstrated with both full-wave simulations and measurements. The simulated absorptivity exceeds 91% around 10.45 GHz at an angle of incidence up to 70° in both TM and TE polarizations. The measured absorptivity at 10.45 GHz is close to 96.5% for all polarization angles under normal incidence. As the angle of incidence changes from 0° to 70°, the measured absorptivity at 10.45 GHz remains above 90% in the TE mode and higher than 94% in the TM mode. Under an oblique incidence, the measured 90% absorption bandwidth is 1.95% from 10.1–10.2 GHz and 10.4–10.5 GHz up to 70° at the TE mode, and 3.39% from 10.15–10.5 GHz up to 70° at the TM mode.

## Introduction

Metamaterial (MM) have found its interest in research in many applications. One of those is a metamaterial-inspired absorber which introduced by Landy in 2008^1^. They generate electric and magnetic resonances, which are controlled independently to tailor the effective permittivity and permeability, respectively. The impedance of the MM is determined from the effective permittivity and permeability. Therefore, MM absorbers can be designed by manipulating the impedance of the MM for the zero reflection coefficient^2^. MM absorbers have several advantages compared with conventional absorbers such as ferrites^3^, composite material-based absorbers^4^, and Salisbury screen absorbers^5^. For instance, MM absorbers can achieve almost perfect absorptivity despite being much thinner than other conventional absorbers^6^. Functionality or tenability can be added to MM absorbers because of printed-circuit-board (PCB) processing^7^. Several researchers have studied the designing of MM unit cells with polarization and incidence angle-insensitivity. Polarization-independent MM absorbers can be realized through a symmetric unit cell^8–14^. Incidence angle-insensitivity can be achieved by the novel geometry of unit cells, such as a split-ring-cross resonator^15^, four-fold rotational symmetric electric resonator with a cross-printed bottom^16^, subwavelength unit cell in a multilayer^17^, surrounding via array^18^, and circular sector^19^. Nevertheless, the resonance of the MM causes a narrow bandwidth, which is a drawback in general applications. Therefore, various methods to enhance the bandwidth of MM absorbers have been introduced by using sectional asymmetric structures^20^, graphene disks^21^, impedance surface^22–24^, multiple layers^25,26^, multiple resonances^26–33^, and destructive interference^34^. However, it was observed that the absorptivity of previous angle-insensitive MM absorbers degraded at different angles of incidence, and the bandwidth decreased at large oblique incidences^8–39^. Therefore, a wide-incidence, bandwidth-enhanced MM absorber needs to be developed.

In this paper, we propose a novel bandwidth-enhanced and wide-angle-of-incidence MM absorber for both transverse electric (TE) and transverse magnetic (TM) polarizations. To achieve high absorptivity and bandwidth enhancement under wide and oblique incidences with both TE and TM polarizations, a hybrid unit cell is introduced to bring the absorption peaks closer to provide a wide-incidence and enhanced-bandwidth unit cell in the proposed MM absorber. The performance of the proposed absorber is demonstrated under normal and oblique incidences with full-wave simulations and measurements. In addition, the proposed MM absorber is compared with previous MM absorbers.

### Design and simulation of the proposed structure

**Figure 1:**
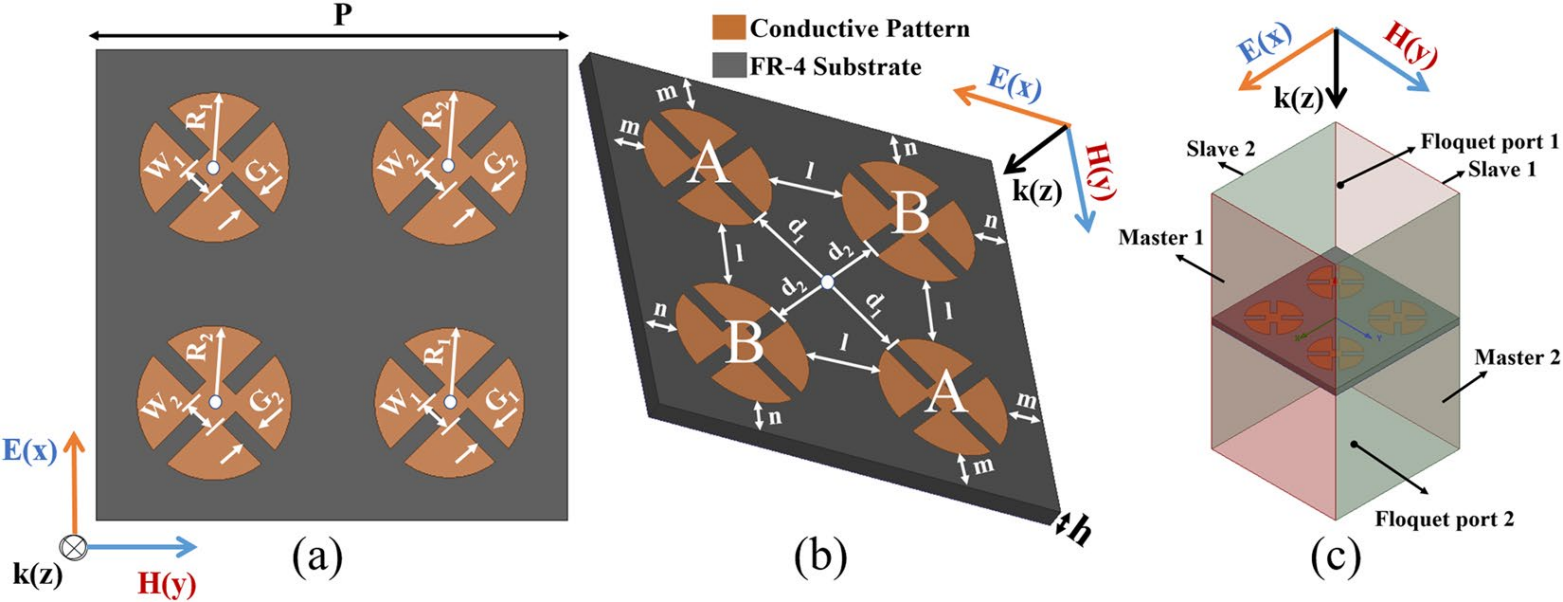
(**a**) Top view, (**b**) 3D view of a unit cell: P = 17.6 mm, R_1_ = 2.8 mm, R_2_ = 2.89 mm, W1 = 1.22 mm, W2 = 1.6 mm, G1 = 0.7 mm, G2 = 0.7 mm, h = 0.8 mm, l = 3.12 mm, m = 1.6 mm, n = 1.51 mm, d1 = 3.44 mm, and d2 = 3.32 mm, (**c**) boundary and excitation setup for EM simulation.

The circular-sector metallic patch structure has recently been employed in the design of electromagnetic (EM) absorbers^19^. The proposed MM absorber is illustrated in Fig. 1. We used an FR-4 substrate with a thickness of h = 0.8 mm. Its relative permittivity and dielectric loss tangent are 3.9 and 0.02, respectively. In general, FR-4 substrates are not used for the X-band, because of their high dielectric loss. However, FR-4 substrates are good candidates for absorber applications because of their high dielectric loss and low cost. The absorptivity *A*(*ω*) can be calculated as follows:

1 $$A(\omega )=1\,-\,{\rm{\Gamma }}(\omega )\,-\,T(\omega )$$

An MM absorber can achieve high absorptivity when the reflection coefficient Γ(*ω*) and transmission coefficient *T*(*ω*) are zero. Under normal incidence, the reflection coefficient can be obtained as

2 $${\rm{\Gamma }}(\omega )=\frac{Z(\omega )\,-\,{Z}_{0}}{Z(\omega )+{Z}_{0}}$$

where *Z*(*ω*) and *Z*_0_ are the impedances of the MM absorber and free space, respectively. Therefore, the zero-reflection condition is satisfied when *Z*(*ω*) = *Z*_0_ = 377 Ω. The intrinsic impedance *Z*(*ω*) of the absorber depends on the effective permittivity (*ε*_r_) and permeability (*μ*_r_):

3 $$Z(\omega )=\sqrt{\frac{{\mu }_{0}{\mu }_{r}(\omega )}{{\varepsilon }_{0}{\varepsilon }_{r}(\omega )}}$$

when *Z*(*ω*) is identical to *Z*_0_, the reflection coefficient becomes zero according to equation (2). Therefore, we can achieve a reflection coefficient of zero by tailoring *ε*_*r*_ and *μ*_*r*_ to be identical each other.

If the reflection coefficient becomes zero, there is no reflected wave. While the bottom plane is fully covered by the conductor, the transmission coefficient *T*(*ω*) is zero and the transmitted EM wave is dissipated from dielectric losses. In general, high-loss dielectric materials are required for broad bandwidth and lower thickness. However, low-loss dielectric materials are preferred for sensing or imaging applications because of selectivity^19,35^.

Under normal incidence, the reflection coefficient can be controlled by manipulating the effective permittivity and permeability of the absorber. However, the reflection coefficient changes for parallel (Γ_∥_) and perpendicular polarizations (Γ_⊥_).

4 $${{\rm{\Gamma }}}_{\perp }(\omega )=\frac{Z(\omega )cos{\theta }_{i}\,-\,{Z}_{0}cos{\theta }_{t}}{Z(\omega )cos{\theta }_{i}+{Z}_{0}cos{\theta }_{t}},$$

5 $${{\rm{\Gamma }}}_{| | }(\omega )=\frac{Z(\omega )cos{\theta }_{t}\,-\,{Z}_{0}cos{\theta }_{i}}{Z(\omega )cos{\theta }_{t}+{Z}_{0}cos{\theta }_{i}},$$

where *θ*_*i*_ and *θ*_*t*_ are the incidence and transmission angles, respectively. It is difficult to minimize the reflection coefficient because it can change under various conditions, such as in equations (2), (4), and (5). Therefore, a wide-incidence, bandwidth-enhanced MM absorber needs to be developed.

Figure 1 displays the top and three-dimensional (3D) views of the proposed unit cell. The top of the unit cell consists of a periodic unit cell, which is composed of four patches of circular-sector metallic plates, while the bottom layer is fully covered with a copper sheet. As shown in Fig. 1, the hybrid unit cell is designed from four patches. Each patch was studied in the previously published our work^19^. Since the unit cell is horizontally and vertically symmetric, its absorptivity is expected to be identical for all polarization angles (*ϕ*). The single unit cell is angle- and polarization-insensitive^32^. In this study, the structure is further modified and a 2 × 2 array is proposed, where the first and fourth structures are identical (denoted as A), and the second and third structures are identical (denoted as B), as shown in Fig. 1(a) and (b). These two different sets of resonating structures will give rise to two distinct absorption peaks. Instead of scaling the whole unit cell structure, some of the geometric dimensions of the structures (W_1_, W_2_; G_1_, G_2_; R_1_, R_2_) are optimized to bring the peaks closer and realize a bandwidth-enhanced absorber.

The final dimensions of the proposed unit cell after optimizing the parameters, as shown in Fig. 1, are P = 17.6 mm, R_1_ = 2.8 mm, R_2_ = 2.89 mm, W_1_ = 1.22 mm, W_2_ = 1.6 mm, G_1_ = 0.7 mm, G_2_ = 0.7 mm, h = 0.8 mm, l = 3.12 mm, m = 1.6 mm, n = 1.51 mm, d_1_ = 3.44 mm, and d_2_ = 3.32 mm.

**Figure 2:**
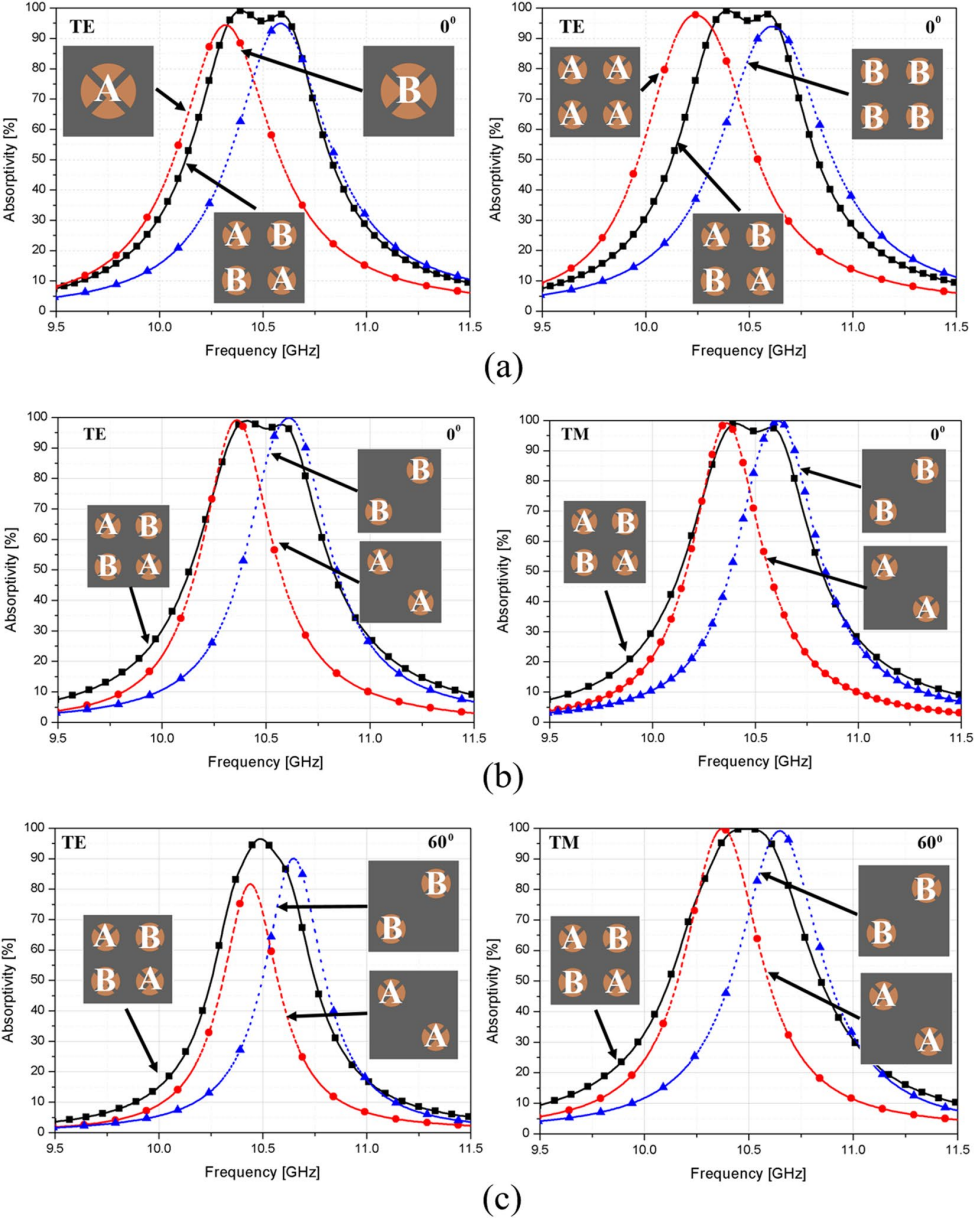
Simulated results for both resonator frequencies. They are sufficiently close to each other to appear as a single resonance absorber with an enhanced bandwidth using hybrid unit cells in a 2 × 2 array: (**a**) TE and (**b**) TM modes under normal incidence θ = 0° and (**c**) oblique incidence θ = 60°.

Based on the single patch using the circular sector^19^, a wide angle of incidence-insensitivity is exhibited. We proposed a method to enhance the bandwidth by using hybrid unit cells. In the design, the first resonant frequency is proposed where the first and fourth patches are identical (denoted as A in Fig. 1b), and the second resonant frequency is proposed where the second and third patches are identical (denoted as B in Fig. 1b). By using the proposed approach of having the resonators oriented in a 2 × 2 array, we present the simulation results in Fig. 2, which shows how bandwidth broadening is achieved. In Fig. 2a, unit cells with a single patch—A or B—and unit cells with four patches—AAAA or BBBB—were investigated. However, all of them had a 90% absorption bandwidth approximately 1.95% narrower than the unit cell using the ABBA hybrid unit cells with a 90% absorption bandwidth of 3.82%. In this method, we wanted to enhance the bandwidth by using the resonant frequencies, which were very close to each other. Thus, we could combine patch A as the lower resonant frequency and patch B as the higher resonant frequency. However, the polarization-insensitivity requires a symmetric structure.

To design a symmetric structure, we proposed hybrid unit cells, as shown in Fig. 2(b) and (c). The first and fourth subcells are responsible for the absorption at lower frequencies, whereas the higher resonant frequency is more pronounced in the second and third structures. Since the resonant frequencies of these structures are very close to each other, these absorption peaks result in bandwidth enhancement. Moreover, in Fig. 2(b) and (c), it can be observed that the combination of the two resonances results in perfect absorption, although individually, the absorption levels of the resonators are not perfect. The proposed absorber designed using hybrid unit cells indicates that a large enhancement in bandwidth is possible using dual close-resonant frequencies.

**Figure 3:**
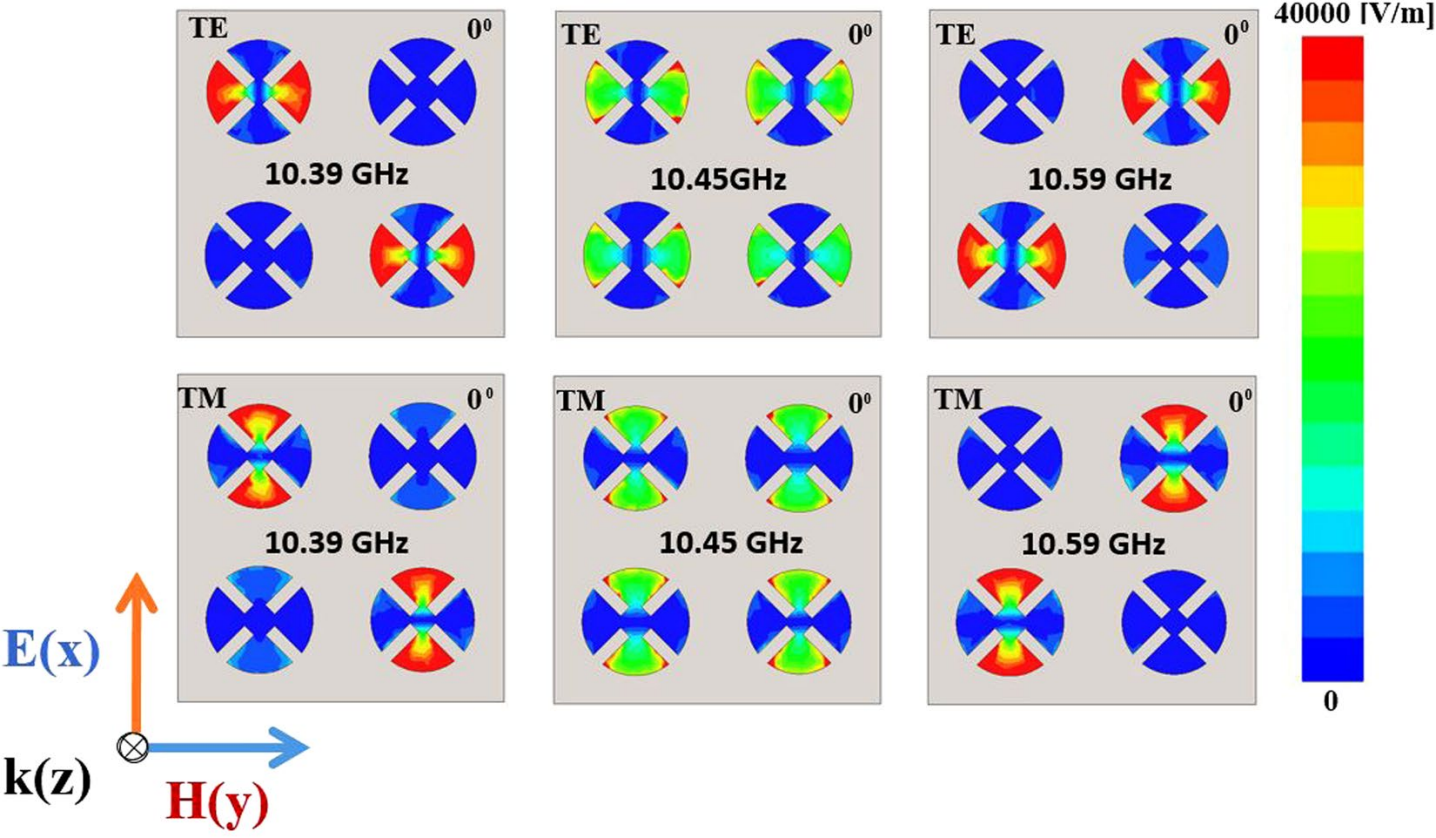
Magnitude of the electric field distribution of the hybrid unit cells at 10.39 GHz, 10.45 GHz (centre frequency), and 10.59 GHz at the TE and TM modes under normal incidence (*θ* = 0°).

**Figure 4:**
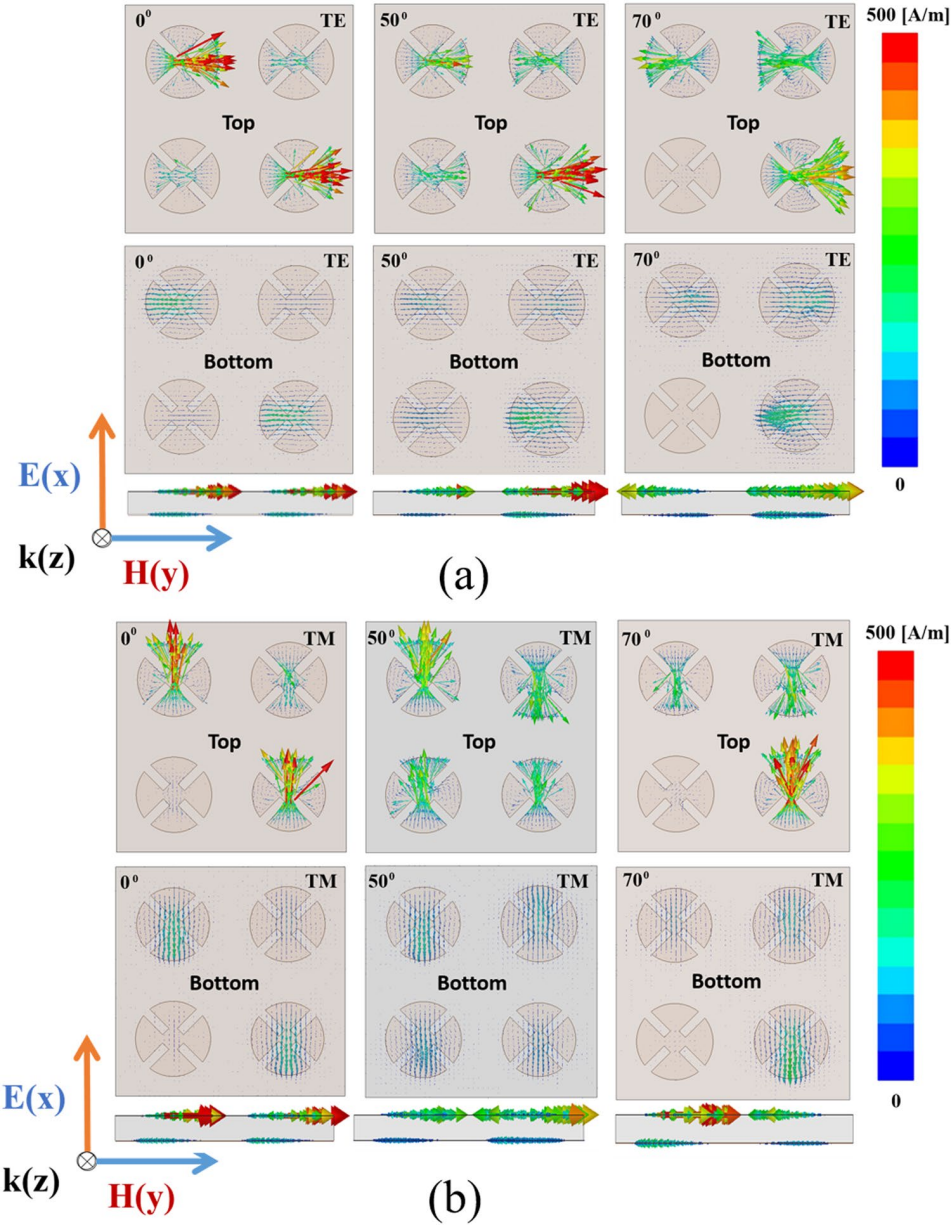
Simulated vector-current distribution of the top, bottom, and side of the hybrid unit cells at the centre frequency of 10.45 GHz. (**a**) TE mode and (**b**) TM mode under normal incidence (*θ* = 0°) and oblique incidence (*θ* = 50° and 70°).

Figure 3 shows the simulated magnitude of the electric field distributions on the XY plane of the hybrid unit cells at the first resonant frequency of 10.39 GHz, a centre frequency of 10.45 GHz, and the second resonant frequency of 10.59 GHz under normal incidence (θ = 0°) for both TE and TM modes. The electric field distribution at 10.39 GHz, shown in Fig. 3, is due to the effect of patches A-A, that at the centre frequency is due to the effect of all patches AABB, and the second resonant frequency of 10.59 GHz is strongly affected by the patches BB. The MM absorber can be understood in terms of electric and magnetic resonances, and visualized by plotting the electric field magnitude and vector-current distributions^18,19^. It is observed that the electric field is strong for the hybrid unit cells of the 2 × 2 array. In addition, the antiparallel currents at the top and bottom of the unit cell under normal incidence (θ = 0°) and oblique incidences (θ = 50 and 70°) for both TE and TM modes are shown in Fig. 4(a) and (b), respectively. The circulating and antiparallel currents are responsible for the magnetic resonance. Owing to the high loss from the dielectric material at the resonant frequency, the magnitude of the electric current in the bottom plane is weaker than that in the top plane. At the centre frequency of 10.45 GHz, the top and bottom surface currents are antiparallel, which constitutes a circulating loop around the incident magnetic field and generates magnetic excitation. Conversely, the top surface metallic-patch array is electrically excited by the incident electric field. Both excitations become significant at the absorption frequencies, and near-unity absorption is realized. Moreover, the antiparallel currents form a magnetic dipole, which functions as a circulating current. The direction of the magnetic dipole is along the incident magnetic field polarization. Therefore, it strongly traps the incident magnetic energy, thus resulting in minimum reflection and strong absorption within the lossy dielectric material.

**Figure 5:**
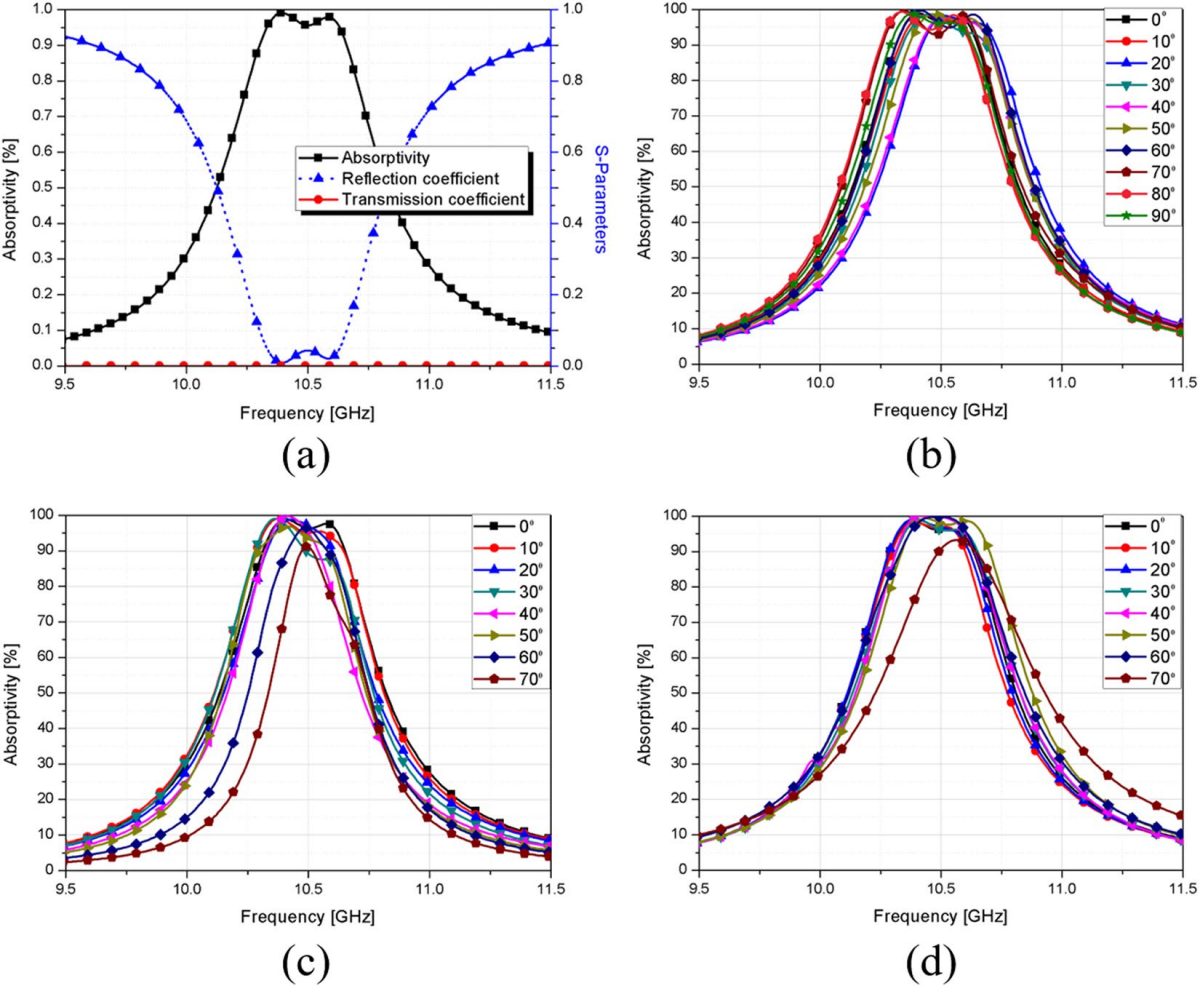
(**a**) Simulated reflection coefficient, transmission coefficient, and absorptivity of the proposed MM absorber under normal incidence. (**b**) Simulated absorptivity of the proposed absorber for polarization angles (*ϕ*) ranging from 0–90°. Simulated absorptivity of the proposed MM absorber for angles of incidence (*θ*) ranging from 0–70° at the (**c**) TE mode and (**d**) TM mode.

Figure 5(a) shows that the proposed absorber can achieve high absorptivity under normal incidence because the reflection coefficient *Γ*(*ω*) and transmission coefficient *T*(*ω*) are zero at the resonant frequency. The bandwidth-enhanced structure is also polarization-insensitive, as observed in Fig. 5(b). Owing to the structural symmetry, the simulated absorptivity is 96% at the centre resonant frequency of 10.45 GHz for all polarization angles (*ϕ*) ranging from 0–90°. In Fig. 5(c) and (d), the resonant frequency does not change until *θ* is 70°, and the absorptivity is maintained higher than 90% till 70° for the TM and TE modes.

### Fabrication and measurement results

**Figure 6:**
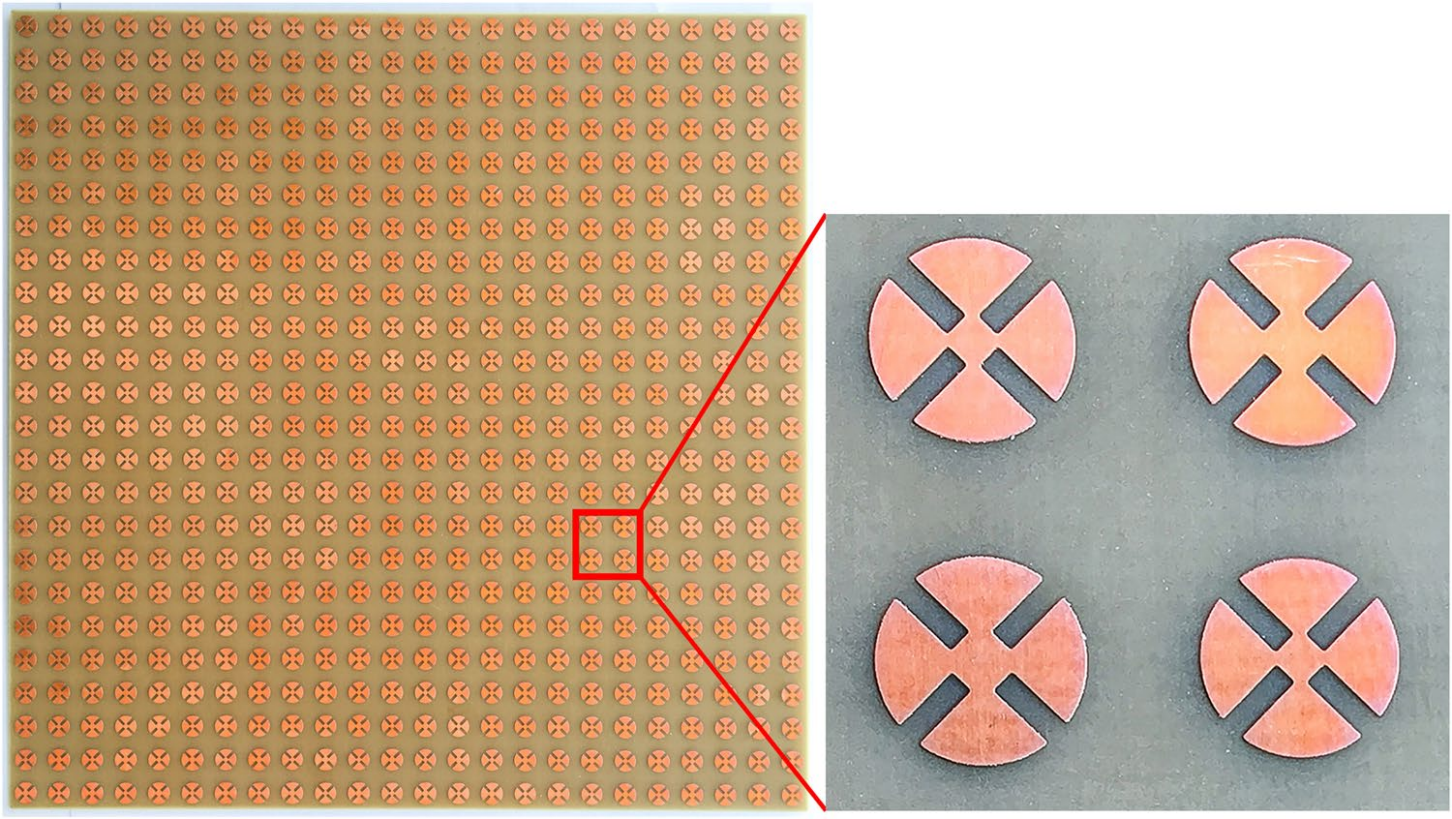
Images of the fabricated (**a**) MM absorber prototype and (**b**) magnified hybrid unit cells.

**Figure 7:**
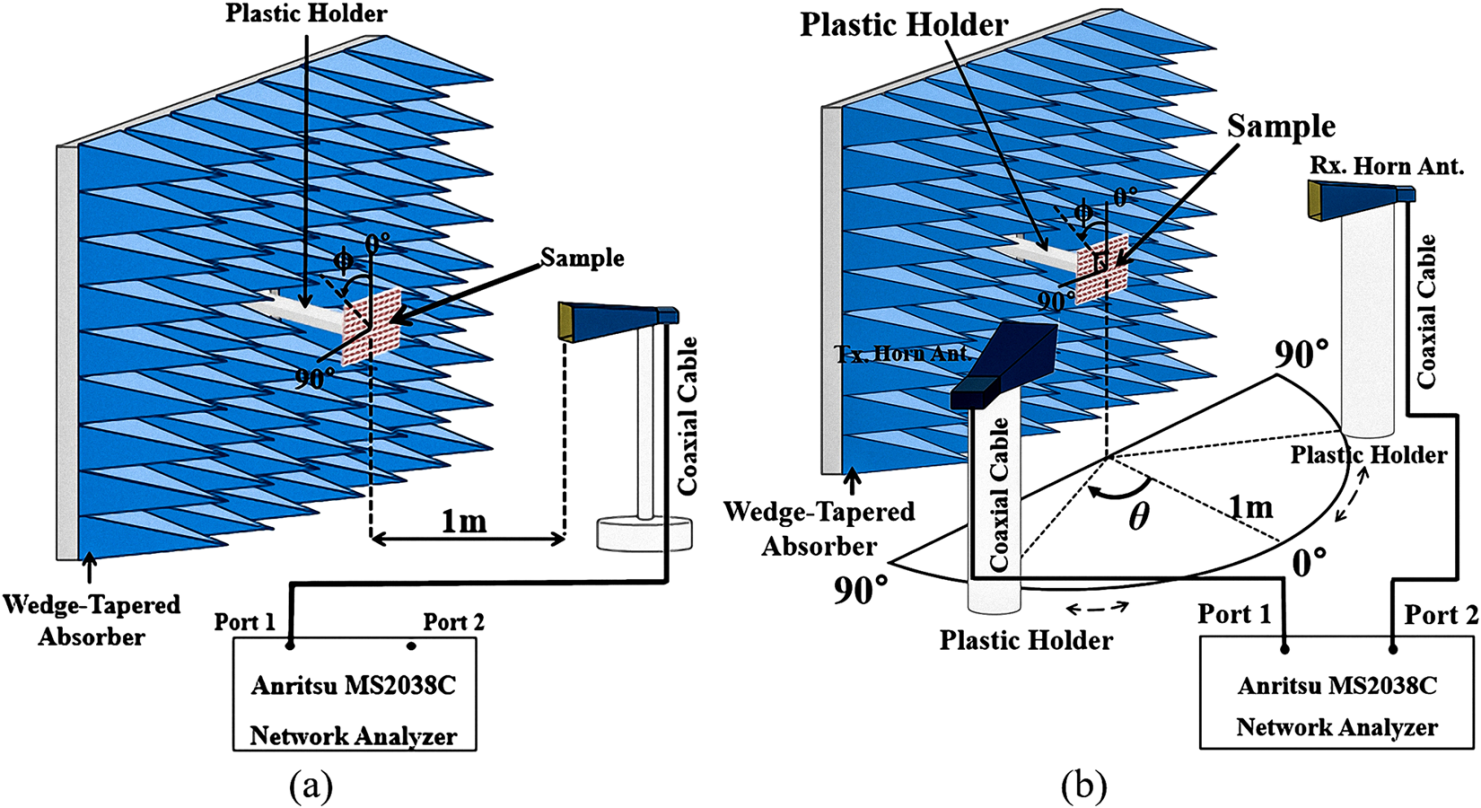
Illustration of the free-space measurement setup for (**a**) normal incidence and (**b**) oblique incidence.

The wide-incidence, bandwidth-enhanced MM absorber using hybrid unit cells shown in Fig. 1 is fabricated on an FR-4 substrate with the prototype 12 × 12 unit cells. Figure 6 displays an image of the fabricated prototype with an overall size of 212 × 212 mm^2^. We used copper for the conductive patterns on the top and bottom planes. The setup of the measurement environment is illustrated in Fig. 7. We used an Anritsu MS2038C vector network analyzer (VNA) to measure the S-parameters in free space. For transmission and reception, a Pasternack PE9856/SF-15 WR-90 standard-gain horn antenna is used. Its nominal gain is 15 dB. The vertical and horizontal 3-dB beamwidths are 29° and 29.3°, respectively. Since the infinite periodic array is simulated, a larger sample size is preferred. However, the sample size had to be limited due to fabrication limitations. We verified that the measurement results with the experimental setup in Fig. 7 were similar to the simulation results when the sample size was larger than 150 mm × 150 mm. According to equation (1), the absorptivity is derived from the reflection coefficient and transmission coefficient. We used the single horn antenna to measure the absorptivity under normal incidence. We observed that the measured absorptivity was 96.5% around the centre frequency of 10.45 GHz, while the simulated absorptivity was 96% at 10.45 GHz. Thus, the simulated and measured results showed the same resonant frequency.

**Figure 8:**
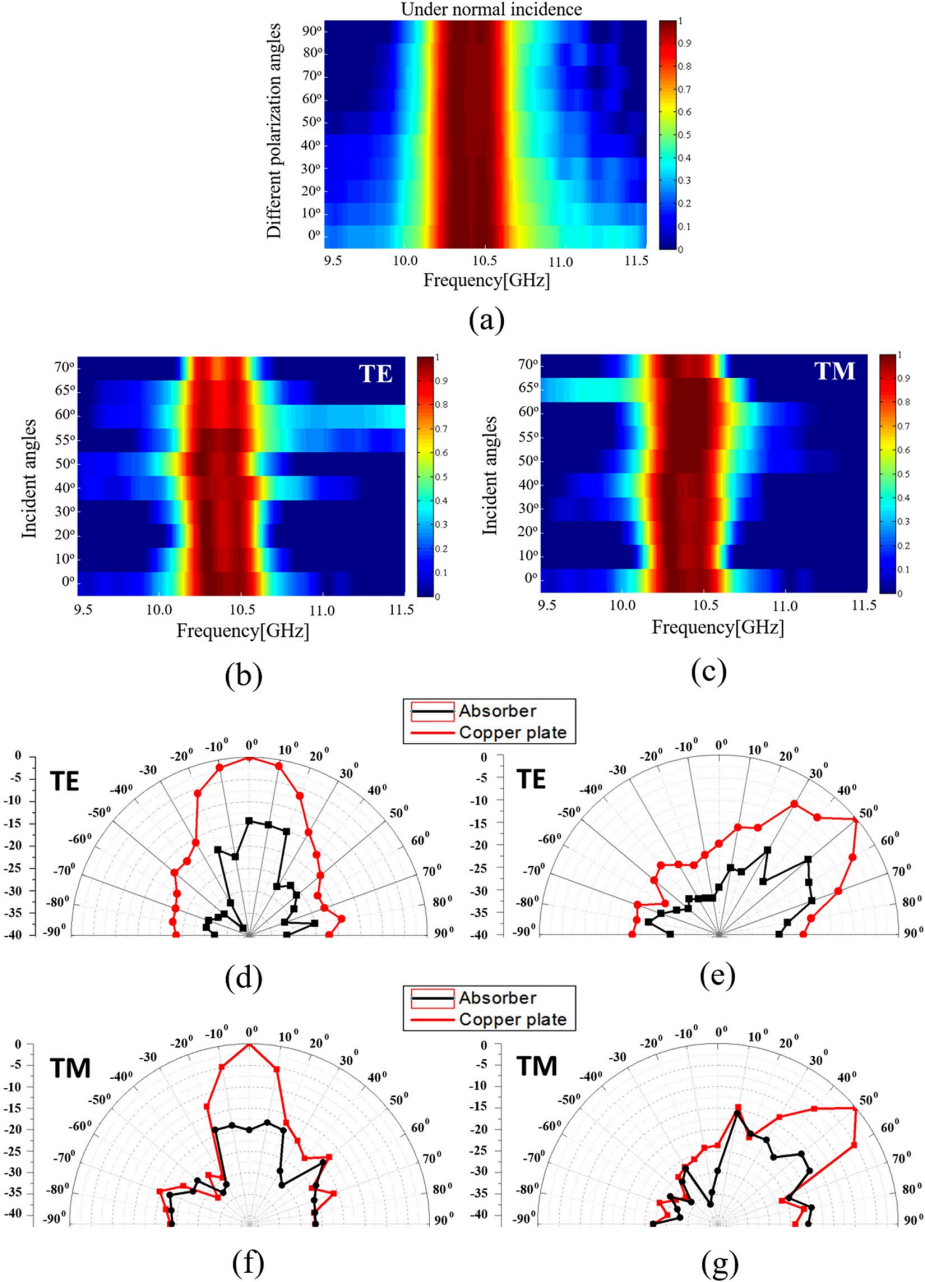
(**a**) Measured absorptivity at the specular angle of the proposed absorber for different polarization angles (ϕ) under normal incidence ranging from 0–90°. Measured absorptivity at the specular angle of the proposed absorber for angles of incidence (*θ*) ranging from 0–70° in (**b**) TE and (**c**) TM polarizations. Bistatic RCS measurement results at 10.45 GHz for the TE mode under (**d**) normal incidence (*θ* = 0°) and (**e**) oblique incidence (*θ* = 50°). For the TM mode under (**f**) normal incidence (*θ* = 0°) and (**g**) oblique incidence (*θ* = 50°).

To observe the polarization sensitivity of the proposed absorber, the horn antenna was rotated for a polarization angle (*ϕ*) ranging from 0–90° under normal incidence. Figure 8(a) shows the measured absorptivity at the specular angle of the fabricated prototype at different polarization angles. It was successfully demonstrated that the absorptivity and frequency of the proposed absorber do not change for all polarization angles.

We used two horn antennas to measure the absorptivity at the specular angle under oblique incidence. One antenna was used to transmit EM energy, and its angle of incidence was changed by rotating the horn antenna from 0° to 70°. The other horn antenna was used to receive the EM energy reflected from the absorber, and it was placed at an angle to satisfy Snell’s law. Figure 8(b) and (c) show the measured absorptivity at the specular angle of the fabricated prototype at the TE and TM polarizations, respectively. As shown in Fig. 7, the wedge-tapered absorbing materials are placed only on the backside of the fabricated sample and the measurement setup is optimized for normal incidence measurement. Therefore, the measurement error is unavoidable for oblique incidence. For the TM polarization, the absorptivity and resonant frequency remain unchanged up to *θ* = 70° while maintaining an absorptivity of approximately 94% at the specular angle, at the centre frequency of 10.45 GHz. For the TE polarization, the absorptivity at the specular angle decreases with the angle of incidence. However, an absorptivity greater than 90% is maintained up to *θ* = 70° at the centre frequency of 10.45 GHz. For both the TE and TM modes, the 90% bandwidth absorption frequency ranges from 10.1–10.6 GHz. The simulation and measurement results for different incident angles are compared in Table 1. The measured absorption frequencies are slightly lower than the simulation frequencies because of dielectric constant and fabrication errors. Thus, we successfully demonstrated that an MM absorber using hybrid unit cells could achieve an absorptivity that was insensitive to the angle of incidence of both TE and TM polarizations. Moreover, the hybrid unit cells in the 2 × 2 array achieved an enhanced bandwidth.

**Table 1:** Comparison of the simulated and measured 90% absorption bandwidth under oblique incidence.

90% absorption bandwidth [GHz]								
	*θ* = 0°		*θ* = 30°		*θ* = 60°		*θ* = 70°	
	TE	TM	TE	TM	TE	TM	TE	TM
Simulation	10.25–10.65	10.25–10.65	10.25–10.60	10.25–10.65	10.35–10.60	10.30–10.65	10.45–10.55	10.45–10.68
Measurement	10.15–10.60	10.15–10.60	10.15–10.55	10.15–10.60	10.1–10.50	10.10–10.50	10.20–10.50	10.15–10.50

To verify the measurement results^37^, Fig. 8(d), (e), (f), and (g) present the bistatic radar cross-section (RCS) measurement results of the copper plate and absorber prototype at the centre frequency of 10.45 GHz. The measured bistatic RCSs under the angles of incidence *θ* = 0° and 50° are plotted in Fig. 8(d) and (e) for the TE mode and Fig. 8(f) and (g) for the TM mode, respectively.

It was observed that both the copper plate and MM absorber were specularly reflective. The scattering wave is observed in other angles because of measurement error. It is difficult to measure bistatic RCS in the lab environment because of large physical size of the transmitting and receiving horn antennas. Moreover, the measurement setup is optimized for normal incidence measurement such as one side of the wedge-tapered absorbing materials. Therefore, measurement error is unavoidable for oblique incidence.

We collected the reflective energy across the whole solid angle and accurately obtained the reflectivity for the angles of incidence *θ* = 0° and 50°. The loss power can be obtained from

6 $${P}_{(\theta )Loss}[ \% ]=(1\,-\,\sum _{-9{0}^{\circ }}^{9{0}^{\circ }}\frac{{P}_{A}}{{P}_{C}})\times 100,$$

where *P*_*C*_, *P*_*A*_, and *P*_*(ϑ)Loss*_ are the collected powers of the copper plate, MM absorber, and the total loss power, respectively. For each solid angle (*ϑ*), we fixed one horn antenna, and the second horn antenna collected all reflection coefficients when it moved from −90° to 90°. Based on the bistatic RCS measurement results and equation (6), we can derive the loss power [%] (or absorptivity) of P_TE_(*θ* = 0°)_loss_ = 95.59%, P_TE_(*θ* = 50°)_loss_ = 93.96%, P_TM_(*θ* = 0°)_loss_ = 94.2% and P_TM_(*θ* = 50°)_loss_ = 92.56%. From the loss power values at *ϑ = *0° and 50°, the absorptivities at the specular angles *θ* = 0° and 50°, as shown in Fig. 8(b) and (c) are in good agreement.

## Discussion

In this paper, we proposed an MM absorber using a hybrid-unit-cell design of a 2 × 2 array. To provide an experimental demonstration, the MM absorber was fabricated on an FR-4 substrate consisting of 12 × 12 unit cells. Under normal incidence, the absorptivity of the proposed MM absorber exceeded 96.5% at the centre frequency of 10.45 GHz even for polarization angles (*ϕ*) ranging from 0–90°. Under the oblique incidence of the TE mode, the absorptivity at the centre frequency of 10.45 GHz exceeded 90% even for angles of incidence (*θ*) varying from 0–70°. Under the oblique incidence of the TM mode, the absorptivity exceeded 94% despite the angle of incidence varying from 0° to 70°. For both TE and TM modes, the 90% bandwidth absorption frequency was 3.39% from 10.15–10.5 GHz up to 70°. Thus, the full-wave simulation and measurement successfully demonstrated the proposed MM absorber using hybrid unit cells, the polarization and angular insensitivities, as well as the enhanced bandwidth.

In Table 2, we compare the proposed MM absorber with the MM absorbers that have wide incidences^17–19^. In particular, the proposed MM absorber achieves values higher than 90% for angles of incidence up to 70° for both TE and TM polarizations. Moreover, at wide angles of incidence up to 60° or 70°, the 90% absorption bandwidth of the proposed absorber is 3.39%, whereas the other absorbers^20–25^ exhibit an approximate 90% absorption bandwidth of 0%. Only one reference^18^ has reported a 90% absorption bandwidth of 1.78%, which is still 1.6% less than the proposed absorber.

**Table 2:** Comparison of the performances of the proposed metamaterial absorber and other angle-insensitive metamaterial absorbers at the specular angle.

Ref	Centre Frequency [GHz]	Thickness (mm)	*θ* = 0°		*θ* = 60°		*θ* = 70°	
			BW [%]		BW [%]		BW [%]	
			TE	TM	TE	TM	TE	TM
17	10.28	0.56 (0.019 λ)	1.94 (10.2–10.4)	1.94 (10.2–10.4)	N/A	1.45(10.25–10.4)	N/A	N/A
18	11.3	0.35 (0.013 λ)	2.64 (11.2–11.5)	2.64 (11.2–11.5)	N/A	2.64 (11.2–11.5)	N/A	1.78(11.15–11.35)
19	10.44	0.60 (0.02 λ)	2.87 (10.3–10.6)	2.87 (10.3–10.6)	N/A	0.95 (10.5–10.6)	N/A	N/A
20	4.8 × 10^4^	1.9 × 10^−3^ (0.03 λ)	8.33 (4.6–5 THz)	4.26 (4.6–4.8 THz)	N/A	N/A	N/A	N/A
21	1 × 10^4^	50 × 10^−3^ (0.17 λ)	100 (0.5–1.5 THz)	100 (0.5–1.5 THz)	66.67 (0.5–1 THz)	N/A	N/A	N/A
22	10.5	3.6 (0.126 λ)	66.67 (7–14)	66.67 (7–14)	N/A	N/A	N/A	N/A
23	10.87	5.60 (0.2 λ)	75.13 (6.79–14.96)	75.13 (6.79–14.96)	N/A	N/A	N/A	N/A
24	8.10	4.47 (0.12 λ)	71.6 (5.2–11)	71.6 (5.2–11)	N/A	26.67 (6.5–8.5)	N/A	N/A
25	0.9 × 10^4^	12 × 10^−3^ (0.028 λ)	11.11 (0.85–0.95)	11.11 (0.85–0.95)	N/A	N/A	N/A	N/A
This Work	10.45	0.80 (0.028 λ)	4.34 (10.15–10.6)	4.34 (10.15–10.6)	3.88 (10.1–10.5)	3.88 (10.1–10.5)	1.95 (10.1–10.2) & (10.4–10.5)	3.39(10.15–10.5)

*N/A: Not applicable. *BW [%]: 90% absorption bandwidth [%].

## Methods

### Simulation

To simulate the performances of the proposed absorber, we used a finite-element-method-based ANSYS high-frequency structure simulator (HFSS). Since the proposed MM absorber is a periodic structure, the master/slave pair was used for the boundary condition. As shown in Fig. 1(c), two YZ-planes are assigned as a master 1/slave 1 pair. Similarly, two XZ-planes are assigned as a master 2/slave 2 pair. The Floquet port is used as the excitation port setup. As shown in Fig. 1(c), two XY-planes are excited as ports 1 and 2, respectively. The air box size is 17.6 × 17.6 × 30.8 mm^3^, where the height above and below the unit cell is 15 mm and 15 mm, respectively. The conductivity of copper is set as 58 × 10^6^ S/m. The dielectric constant and tangential loss of the FR-4 substrate are set as 3.9 and 0.02, respectively. Since the polarization angle (φ) and angle of incidence (θ) are respectively defined as “phi” and “theta” on the Floquet port, we simulated the absorptivity for each φ and θ by varying “phi” and “theta”, respectively, on the Floquet port. We simulated the S-parameters with a solution setup as maximum number of passes (30), maximum delta S per pass (0.02), maximum refinement per pass (30%), minimum converged passes as 1, and the default port accuracy (2%). In addition to the S-parameters, we plotted the magnitudes of the electric fields and vector electric current densities using the ANSYS HFSS.

### Measurement

We measured the absorptivity of the fabricated MM absorber in free space, as illustrated in Fig. 7. The absorptivity was derived from the S-parameters, which were measured using an Anritsu MS2038C VNA. We used a single horn antenna to measure S_11_ under normal incidence, as shown in Fig. 7(a), and used two horn antennas to measure S_21_ under oblique incidence, as shown in Fig. 7(b). To allow the EM waves to radiate sufficiently and to minimize the near-field effects, the distance between the antennas and the sample is was kept at 1 m (approximately 34 times larger than the absorption wavelength), which was farther than the far-field of the antenna in the microwave anechoic chamber. The apertures of the two horn antennas and the distance from sample to the middle point of two horn antennas were finely established for incident angles of 0–70°. In addition, the absorber prototype was surrounded by wedge-tapered absorbing materials to remove unwanted reflected and scattered EM waves. To receive the reflected EM wave only from the absorber prototype, we applied the time-gating function of the VNA. In addition, the measurement setup was calibrated with the copper plate. Before measuring the S-parameters of the absorber prototype, we first measured the S-parameter of the copper plate with the same size of the absorber sample. Next, we set the magnitude of its reflection coefficient to 1 for the calibration process^18,19^. To measure the absorptivity at different polarization angles, we rotated the horn antenna from 0° to 90° while fixing its location at *θ* = 0°, and measured the S-parameters at each polarization angle. To measure the absorptivity at different angles of incidence, the transmitting horn antenna was rotated from 0° to 70° on the azimuth plane. At each angle of incidence *θ*, we placed the receiving horn antenna at that angle to satisfy Snell’s law, and measured the S-parameters at each angle of incidence.
